# Molecular Mechanisms That Contribute to Bone Marrow Pain

**DOI:** 10.3389/fneur.2017.00458

**Published:** 2017-09-11

**Authors:** Jason J. Ivanusic

**Affiliations:** ^1^Department of Anatomy and Neuroscience, University of Melbourne, Melbourne, VIC, Australia

**Keywords:** bone marrow, skeletal pain, bone pain, pain, nociception, bone, peripheral, nociceptor

## Abstract

Pain associated a bony pathology puts a significant burden on individuals, society, and the health-care systems worldwide. Pathology that involves the bone marrow activates sensory nerve terminal endings of peripheral bone marrow nociceptors, and is the likely trigger for pain. This review presents our current understanding of how bone marrow nociceptors are influenced by noxious stimuli presented in pathology associated with bone marrow. A number of ion channels and receptors are emerging as important modulators of the activity of peripheral bone marrow nociceptors. Nerve growth factor (NGF) sequestration has been trialed for the management of inflammatory bone pain (osteoarthritis), and there is significant evidence for interaction of NGF with bone marrow nociceptors. Activation of transient receptor potential cation channel subfamily V member 1 sensitizes bone marrow nociceptors and could contribute to increased sensitivity of patients to noxious stimuli in various bony pathologies. Acid-sensing ion channels sense changes to tissue pH in the bone marrow microenvironment and could be targeted to treat pathology that involves acidosis of the bone marrow. Piezo2 is a mechanically gated ion channel that has recently been reported to be expressed by most myelinated bone marrow nociceptors and might be a target for treatments directed against mechanically induced bone pain. These ion channels and receptors could be useful targets for the development of peripherally acting drugs to treat pain of bony origin.

## Introduction

Pain is associated a number of different bony pathologies or disease and puts a significant burden (both in terms of quality of life and cost) on individuals, society, and the health-care systems worldwide (1, 2). For example, nearly 10% of men and 20% of women over the age of 60 years have symptomatic osteoarthritis. Over 50% of postmenopausal white women in northern parts of the USA are estimated to have osteopenia and a further 30% to have osteoporosis. Metastatic bone pain is the most common pain syndrome reported in cancer patients, and up to 50% of patients report the pain being poorly managed by present treatments (2). This burden is expected to increase with advances in modern medicine that prolong life expectancy, because many of the conditions that cause bone pain are intractable and develop late in life. Pain is the major reason why these patients present to the clinical environment. Opioids and non-steroidal anti-inflammatory drugs (NSAIDs) are used to treat mild to severe bone pain, but therapeutic use over long periods required to treat chronic or intractable bone pain is limited by undesirable side-effects including sedation, respiratory depression, tolerance, risk of addiction, gastrointestinal effects, and renal toxicity. Long-term use of opioids and NSAIDs in this setting is also contraindicated because of potentially undesirable effects on bone remodeling/healing (3–5), which may further complicate the underlying pathology. A major impediment to the development of alternative strategies to treat bone pain is the significant challenge of experimental access to nociceptors in bony tissue. A better understanding of how these nociceptors transduce and code information about noxious stimuli applied to bone and how this is changed in pathological situations is critical to the development of more targeted and specific therapies to treat bone pain.

This review explores recent advances in our current understanding of mechanisms that generate and maintain bone pain, with a particular focus on the function of peripheral nociceptors that innervate bone marrow and how their molecular phenotype influences their function. A number of ion channels and receptors are now emerging as important modulators of the activity of peripheral bone marrow nociceptors. Identifying these regulators of nerve activity in bone nociceptors and better understanding their role in generation of bone pain could open up avenues for development of tools to selectively manipulate pain originating from bone.

## Pathology or Disease of the Bone Marrow Activates Bone Marrow Nociceptors and Produces Pain

**Figure 1:**
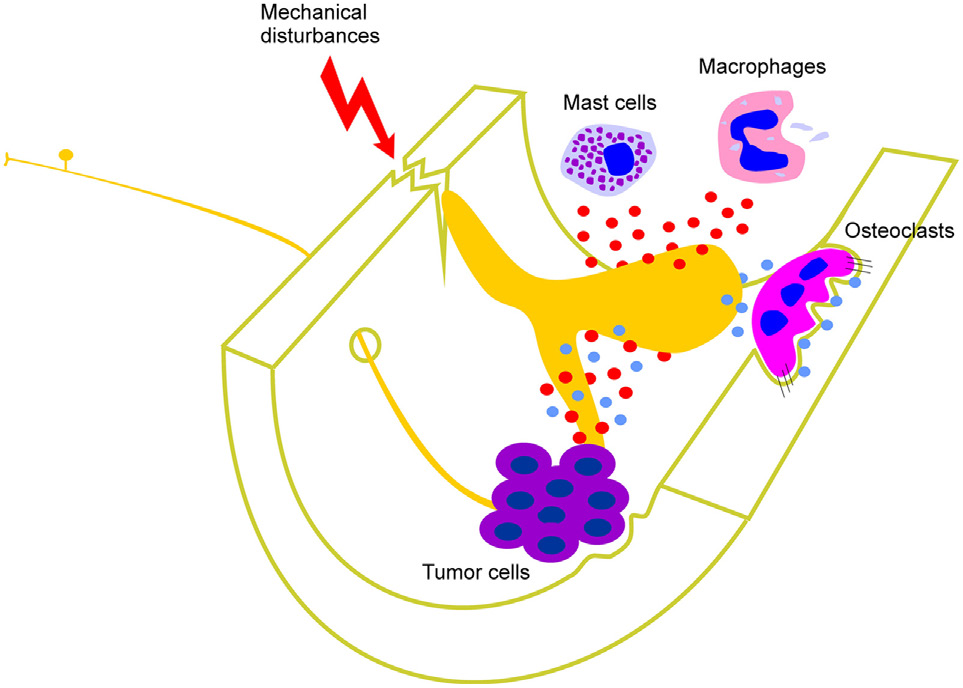
Factors that contribute to activation of peripheral bone marrow nociceptors. Mast cells and macrophages, and cells associated with various different types of tumor in bone, release cytokines, interleukins, growth factors (including NGF), and other inflammatory mediators (represented by red circles). These act directly on the peripheral nerve terminal endings of sensory neurons that innervate the bone marrow. Destruction of bone by osteolytic processes as well as excessive mechanical stress or trauma can also lead to injury or distortion of bone that activates mechanically sensitive bone marrow nociceptors. Osteoclast-mediated bone remodeling and osteolytic tumors are accompanied by the production of extracellular protons (represented by blue circles) which are known to activate nociceptors in other tissues and likely also do so in bone marrow.

Bone cancers, fractures, intraosseous engorgement syndrome, osteoarthritis, and osteomyelitis produce inflammation and/or an increase in intraosseous pressure that can activate peripheral sensory nerve terminals within the bone marrow through the release of inflammatory mediators and/or by mechanical compression or distortion (6–12) (Figure 1). Inflammatory mediators that have been implicated include cytokines, endothelins, growth factors (including NGF), and prostanoids, and most of these have been shown to directly excite nociceptors and contribute significantly to pain profiles in skin, joints, and viscera (13). The role of inflammation is easy to appreciate in conditions such as bone marrow edema and osteomyelitis because these are defined by the presence of inflammation. Osteoarthritis is also now considered an inflammatory disease of subchondral bone (14), and bone cancers also have a significant inflammatory component (15). Destruction of bone by osteolytic processes as well as excessive mechanical stress or trauma can lead to injury or distortion of bone that likely activates mechanically sensory nerve terminals in bone marrow (3, 8, 12) (Figure 1). Agents known to act by reducing inflammatory processes (e.g., NSAIDs and specific COX inhibitors) produce partial analgesia in animal models of cancer-induced bone pain (16–18) and pro-inflammatory cytokines contribute to mechanically induced nociceptive responses in fracture models (19). Osteoclast-mediated bone remodeling is accompanied by the production of extracellular protons (hydrogen ions), which are known to activate nociceptors in other tissues (20–22) (Figure 1). Increased osteoclast activity is a hallmark of osteoporosis (23) and can also occur in some types of bone cancer (24, 25). Bisphosphonates are anti-bone resorption drugs which inhibit osteoclast activity and relieve pain in patients with osteoporosis (26, 27), and in animal models of bone cancer-induced pain (28, 29). Thus, protons released during osteoclast-mediated bone remodeling and/or from osteolytic tumors in the bone marrow are a likely trigger of pain originating in the bone marrow. Taken together, these studies highlight that pathology of the bone marrow is associated with changes that likely activate bone marrow nociceptors and contribute to pain.

## Bone Marrow Nociceptors

In general, pain is transmitted by two main classes of peripheral nociceptors (30). Aδ nociceptors are small-diameter myelinated sensory neurons that transmit fast, intense pain, of the sort experienced in response to fracture, acute inflammation, or mechanical instability of bone. C nociceptors are small-diameter unmyelinated sensory neurons that encode slow, aching pain of the sort experienced in more chronic conditions, such as osteoarthritis or bone cancer. Both Aδ- and C-fiber sensory neurons innervate the bone marrow (31–36), contain molecular markers for nociceptors, such as substance P, calcitonin gene-related peptide (CGRP), tropomyosin receptor kinase A (TrkA), and transient receptor potential cation channel subfamily V member 1 (TRPV1) (33, 35, 37–42), and are responsive to noxious chemical and mechanical stimuli (31, 32, 42–45). Some larger diameter sensory neurons with specialized/encapsulated nerve terminal endings have been reported in the mandibular periosteum of cats (46, 47), human long bone periosteum (48), and Haversian canals in canine cortical bone (49), but not in the bone marrow.

Injection of physiological saline under pressure into the bone marrow of rats leads to activity dependent changes in Fos protein expression in the superficial dorsal horn (50, 51). Fos is used routinely to study activity dependent changes in central neurons that are activated either directly, or indirectly by input from peripheral nociceptors, and the superficial dorsal horn of the spinal cord has a well-established role in processing of nociceptive input (52). Spinal dorsal horn neurons also respond to balloon inflation within the bone marrow of the rat femur (36), and electrical stimulation of nerves that enter the bone marrow of the cat humerus generates evokes potentials in topographically relevant areas of the primary and secondary somatosensory cortices (53). These findings are consistent with a primary role for sensory neurons that innervate the bone marrow in nociception.

While we know a lot about the physiology of sensory neurons that innervate the periosteum, very little is known of those that innervate the bone marrow (54). This is important to consider because almost all bony pathologies involve bone marrow or subchondral bone, and so bone marrow nociceptors are critical to the experience and management of bone pain. Bone marrow is a very difficult medium to work with. It is located deep within the body and is encased by an outer shell of hard compact bone, making experimental access difficult. This has hampered past attempts to study physiological mechanisms of bone pain. Until recently, there were only two published studies of how peripheral sensory neurons respond to noxious stimulation of bone marrow, and both were very limited in scope (31, 32). In these studies, whole-nerve recordings were made from branches of the tibial nerve, while mechanical, thermal, or chemical stimuli were applied to the marrow cavity of anesthetized dogs. Increases in whole-nerve activity were evoked by mechanical and chemical stimulation, but no attempts were made to explore the activity of single units and so it was not clear how individual bone marrow nociceptors respond to different types of noxious stimuli. Nonetheless, both studies showed that an increase in intraosseous pressure of approximately 3–5 times that of normal intraosseous pressure was required to mechanically activate the lowest threshold units in the whole-nerve recordings. The thresholds they reported were between 100 and 130 mmHg. These very high pressures required for activation of bone marrow mechano-receptors are unlikely to be experienced under normal physiological conditions, and are of the order of magnitude experienced by humans in pathological conditions such as intraosseous engorgement syndromes (6, 55). Application of known algesic substances (potassium chloride, acetylcholine, histamine, serotonin, and bradykinin) to the bone marrow in one of these studies also produced an increase in whole-nerve ongoing activity (32). Thermal sensitivity was tested in this same study (32), but only secondary to ischemia produced by experimental ligature and application of vasoconstrictors, so it is not clear if the activity was related to temperature changes or other changes that occurred with ischemia.

More recently, an *in vivo* bone–nerve preparation has been developed in our laboratory to record the activity of bone marrow nociceptors that innervate the rat tibial marrow cavity (42, 45). In these studies, recordings were made from a small nerve, proximal to its entry into bone, in response to noxious mechanical stimulation delivered by increasing intraosseous pressure with injections of isotonic saline. Spike discrimination software was used to isolate single Aδ nociceptors from the whole-nerve recordings. The lowest thresholds for mechanical activation in the whole-nerve recordings were consistent with the reports described above. However, many of the *single* mechanically sensitive Aδ units that were isolated had thresholds for mechanical activation that were significantly greater (up to 230 mmHg). These studies provided evidence that single Aδ bone marrow nociceptors are capable of signaling either the intensity or rate of change in intraosseous pressure (45). It was suggested that those that responded to the intensity of sustained intraosseous pressure may signal pain associated with pathologies that involve sustained increases in pressure within bone, for example intraosseous engorgement syndrome. In contrast, those that responded to changes in the rate of change in intraosseous pressure are likely to signal pain associated with rapid changes in pressure within the marrow cavity, for example, during needle aspiration of bone marrow or emergency intraosseous vascular access. The response of single bone marrow nociceptors with C-fiber conduction velocities has not yet been reported.

Sensitization of peripheral bone nociceptors has been used to explain, in part, the increased sensitivity of patients to mechanical stimuli in various bony pathologies (8, 16, 56). Sensitized peripheral nociceptors are hyper-excitable, that is, they have reduced thresholds for activation and increased activity in response to a given stimulus, making them more sensitive to noxious stimuli, thereby contributing to increased pain sensation (57). These changes are collectively referred to as peripheral sensitization. Peripheral sensitization typically occurs as a result of repeated mechanical or thermal stimulation, or in response to known algesic substances or inflammatory mediators (57). At the cellular level, changes in the distribution and/or function of ion channels and receptors determine peripheral sensitization (57, 58). Much work has focused on the role of transient receptor potential channels, voltage gated sodium channels (VGSCs), and the neurotrophic factor receptors. Post-translation modifications of some of these in peripheral nerve terminals contribute to acute changes in sensitivity and pain (57, 59–61). Long-term changes in their level of expression, driven by transcriptional regulation at the soma of peripheral sensory neurons, contribute to prolonged changes in sensitivity and persistent pain (57, 59, 60, 62, 63).

Evidence of sensitization of peripheral nociceptors has been provided in animal models of bone cancer-induced pain (64, 65). In these studies, the authors showed increased spontaneous activity and reduced heat thresholds for cutaneous C-fiber afferent neurons recorded from the skin around the tumor bearing bone, not of bone afferent neurons. Direct evidence of peripheral sensitization in bone marrow nociceptors has more recently been reported in response to application of at least some algesic substances. After application of capsaicin (a TRPV1 agonist) or nerve growth factor (NGF), some Aδ bone marrow nociceptors had reduced thresholds for activation and increased discharge frequency in response to mechanical stimulation (42, 45). Thus, bone marrow nociceptors can be sensitized to mechanical stimuli and this likely contributes to mechanically induced bone pain.

## NGF and Inflammatory Bone Pain

Nociceptors can be classified into two groups based on their response to NGF or glial cell line-derived neurotrophic factor (66). NGF acts through the p75 and TrkA receptors. The p75 receptor is a 75-kDa protein that binds all neurotrophins with similar affinity. TrkA is a member of the Trk receptor family, which are a group of homologous 140 kDa proteins that bind specific neurotrophins (with high affinity). TrkA confers specificity for NGF. In humans, mutations of the TrkA genes have been reported in individuals that have a congenital insensitivity to pain (67–69). There is an increase in NGF levels in human pain conditions that are characterized by inflammation, such as arthritis (70, 71), and NGF applied exogenously to the human skin or muscle produces hyperalgesia or allodynia (72, 73). In animal studies, TrkA receptor knockout mice are hypoalgesic (74) and transgenic animals overexpressing NGF are hyperalgesic (75). As in humans, NGF is elevated in animal models of acute and chronic pain conditions and NGF sequestration alleviates hyperalgesia in animals (76–80). Importantly, most of these studies have used inflammatory models or examined conditions that have a significant inflammatory component, reinforcing a role for NGF signaling inflammatory pain.

Present evidence clearly indicates a role for NGF in inflammatory pain in various tissues, and this has led to the development of NGF sequestration for management of inflammatory bone pain (81). As detailed above, bone cancers, fractures, osteoarthritis, and osteomyelitis produce inflammation that can activate peripheral bone marrow nociceptors through the release of inflammatory mediators (Figure 1), and these include growth factors such as NGF. Sequestering NGF by systemic administration of anti-NGF antibodies can alleviate, in part, pain-like behaviors in animal models of bone cancer and fracture-induced pain and also inflammatory pain of other tissue systems (82–88). This approach has been successfully applied in clinical trials to manage osteoarthritic pain, but many of the patients receiving the treatment developed rapidly progressive osteoarthritis and required joint replacement (89–93). The problem was so severe that it temporarily halted phase III clinical trials. The halt has now been lifted by the FDA and a number of new trials are under way (94). No attempt is made here to provide an up-to-date review of this literature. Instead, below is provided a review of the mechanisms by which NGF could interact with bone marrow nociceptors to mediate bone pain.

Mantyh and colleagues have demonstrated that TrkA is expressed in a substantial proportion of peripheral nerve terminals in murine long bones (87, 95–97). They used antibodies directed against CGRP and NF200 to identify peptidergic and/or myelinated sensory nerve terminals in bone, respectively. Over 80% of these expressed TrkA. Bone marrow sensory nerve terminals that express TrkA have also been reported in the rat tibia, but in this case they were defined by the absence of coexpression of tyrosine hydroxylase, a marker of sympathetic nerve terminal endings (42). Approximately two-thirds of DRG neurons retrograde labeled from the rat tibia express TrkA and p75 receptors (42), and a similar proportion is reported for DRG neurons projecting to subchondral bone of the rat femur (41). Importantly, the relative proportion of TrkA + sensory neurons that innervate bone is significantly greater than in published reports of TrkA + sensory neurons innervating skin, muscle, joint, and viscera (41, 98, 99), suggesting that NGF signaling may be more important in bone pain than in pain arising from other tissues. Coexpression of TrkA with TRPV1 and Nav1.8, but not Nav1.9, in retrograde labeled bone marrow nociceptor neurons further suggests that TRPV1 and/or Nav 1.8 may contribute to NGF-induced signaling in these neurons (42).

NGF applied directly to the bone marrow rapidly activates and alters the excitability of single mechanically sensitive bone marrow nociceptors in the rat *in vivo* bone–nerve preparation described above (42). The changes in activity reported occur with a very short latency and resolve within 15–30 min, and so the effect of a bolus of locally infused NGF appears to be transient. The same dose of NGF applied to the tibia also alters weight-bearing bearing in the hind limbs with a similar time-course (42). A function blocking anti-TrkA antibody and a mast cell stabilizer was used in this study to show that NGF-induced changes in ongoing activity and mechanical sensitivity are dependent on signaling through the TrkA receptor, but are not affected by the activity of mast cells, respectively. Together, the findings suggest that acute behavioral responses to NGF in bone can be explained at least in part by the rapid activation and/or sensitization of mechanically activated bone marrow nociceptors, and that NGF needs to be present in around nerve terminals in bone for these changes to be maintained. This may explain why sequestering NGF after inflammatory pain has already developed attenuates pain behaviors in animal models of bone cancer and skeletal fracture (100).

Binding of NGF to TrkA on peripheral nerve terminals of peptidergic nociceptors, and the subsequent internalization and retrograde transport of the NGF/TrkA receptor complex (101) results in sensitization of primary afferent neurons that involves upregulated expression of a number of NGF signaling molecules, including VGSCs (62, 63) and TRPV1 (102–104). These changes can in turn increase the excitability of primary afferent neurons and drive persistent changes in the nervous system that are known to occur during inflammatory pain. However, it appears that there is no significant change in the expression of any of these channels subsequent to experimental inflammation of the bone marrow, even at time-points at which animals display significant pain behavior (42). This suggests that sensitization in inflammatory bone pain may not involve long-term changes in protein expression in the soma of bone marrow nociceptors and that retrograde transport of the NGF/TrkA complex and/or upregulation NGF signaling molecules does not appear to be important for the maintenance of persistent pain derived from inflammation in bone.

## The Role of TRPV1 in Sensitization of Bone Marrow Nociceptors

TRPV1 is a non-selective ligand-gated cation channel that integrates many physical and chemical stimuli, including noxious heat (>43°C), protons (pH < 6), capsaicin, and other inflammatory mediators (105). Capsaicin (a TRPV1 agonist) sensitizes peripheral nociceptors to both mechanical and thermal stimulation (106–112). There is an increase in TRPV1 expression in the DRG of animals with cancer-induced bone pain (113–116), and pharmacological blockade of TRPV1 attenuates cancer-induced bone pain (23, 113), suggesting that TRPV1 activation plays a critical role in the generation of at least some types of bone pain.

TRPV1 is expressed in peripheral nerve terminals in the bone marrow (117) and in a substantial proportion (approximately 30%) of retrograde labeled bone marrow nociceptors (42). Capsaicin activates and sensitizes some Aδ-bone marrow nociceptors to mechanical stimulation (45). However, TRPV1 is not thought to transduce mechanical stimuli, and so it is not clear how capsaicin alters mechanical sensitivity in TRPV1 expressing bone marrow nociceptors. Capsaicin-sensitized bone marrow nociceptors have extremely high thresholds for mechanical activation before capsaicin is applied (45). In other tissue systems, nociceptors with these properties are classically described as mechanically insensitive afferents or “silent” nociceptors that under normal conditions are not activated by mechanical stimuli, but after inflammation or chemical stimulation, can become sensitive to a number of different stimulus types, including mechanical stimuli (118, 119).

It is not yet clear if activation of TRPV1 sensitizes bone marrow nociceptors to thermal stimuli. Large changes in the temperature of deep tissues such as bone marrow are not experienced under normal (or even pathological) conditions, and so it is not likely that bone marrow nociceptors could be activated by noxious heat. However, it is possible that activation of the TRPV1 receptor might alter the function of bone marrow nociceptors by making them more sensitive to lower temperatures, and this might be relevant under conditions of inflammation when local tissue temperature increases.

## Acid-Sensing Ion Channels (ASICs) in Bone Pain

Acid-sensing ion channels are voltage-independent proton-gated sodium channels that are activated by a drop in extracellular pH (to pH 5.0) (120). ASICs 1–3 are found in peripheral sensory neurons where they function as homomeric or heteromeric trimers to sense changes in extracellular pH around their sensory nerve terminals (121). There is a particular interest in ASIC 1b and 3 because they are found almost exclusively in DRG neurons. ASICs are upregulated in DRG neurons in animal models of osteoporosis (122) and bone cancer (23). Pathological changes leading to increased bone resorption by osteoclast activation are related to pain-like behaviors in a mouse model of osteoporosis, and inhibiting ASIC3 improves pain-like behavior in this model (122). TRPV1 is also activated by reductions in pH (108, 123) and as noted above, TRPV1 is expressed in bone marrow sensory neurons (42, 117). Blocking TRPV1 improves pain-like behavior in the murine model of osteoporosis (124), but whether this occurs as a result of inhibition of proton mediated activation of TRPV1 is not entirely clear. These findings suggest that acidosis associated with a number of different bony pathologies could be a trigger for pain *via* activation of sensory nerve terminals in bone marrow. However, as both ASICs and TRPV1 are expressed in osteoclasts, blocking these channels might reduce osteoclast mediated changes in bone turnover and pH (125) and thereby indirectly contribute to reduced activation of sensory nerve terminals and pain.

## Pro-Inflammatory Cytokines and Peptides

Several pro-inflammatory cytokines (IL-1β, TNFα, IL-6, and TGFβ) and inflammatory mediators (CGRP) are increased in the DRG in response to bone cancer and fracture (116, 126–132). This suggests that inflammatory mediators may have a role to play in modulating the function of sensory neurons that innervate bone. However, at present there is no evidence that any of these inflammatory mediators directly activate or sensitize bone marrow nociceptors or that changes in their expression alter the function of bone marrow nociceptors.

## Piezo2 and Mechanically Induced Bone Pain

Piezo2 is a newly discovered mechanically gated ion-channel that has received significant attention because of its remarkable structure. It has between 25 and 30 trans-membrane repeats—unlike any other known ion channel (133, 134). Interest in Piezo2 is rapidly advancing now that patients with congenital mutations have been identified and have been shown to have developmental growth defects (135–138). There is significant evidence that Piezo2 is the transducer for low-threshold mechanical stimuli in Merkel cells (139–142) and proprioceptors (143, 144). However, recent evidence suggests Piezo2 might also be involved in the transduction of noxious mechanical stimuli. Mechanically activated Piezo2 currents are enhanced by the algesic peptide bradykinin that drives mechanical hypersensitivity associated with inflammation (145). Furthermore, Piezo2 knockdown in DRG inhibits inflammation-induced mechanical but not thermal hyperalgesia in mouse skin (146) and attenuates viscero-motor pain reflexes in response to noxious and innocuous colorectal distension in rats (147). Piezo2 knockout does not prevent nociceptors from transducing mechanical stimuli but it does selectively reduce the sensitivity of Aδ- (not C-) fiber mechano-nociceptors in the skin–nerve preparation (141). Piezo2 is expressed in myelinated, small-diameter (Aδ) nociceptors that likely mediate responses to noxious mechanical stimulation in the cornea (148, 149) and the majority (70%) of small myelinated (Aδ) nociceptors that innervate the bone marrow express Piezo2 (45). Together, these findings suggest that Piezo2 contributes to the mechanical sensitivity of Aδ mechano-nociceptors, including those that innervate bone marrow. As yet, there are no studies that have directly investigated the functional role of Piezo2 in bone marrow nociceptors, but it is possible that targeting this ion channel might be useful to treat mechanically induced bone pain.

## Conclusion

Pathology that involves the bone marrow triggers pain by activating the sensory nerve terminal endings of peripheral bone marrow nociceptors. A number of ion channels and receptors are now emerging as important modulators of the activity of peripheral bone marrow nociceptors, and these could be useful targets for the development of drugs to treat pain of bony origin.

## Author Contributions

JI was the sole contributor to the conception and design of this review; drafted and revised it critically for important intellectual content; approved the final version to be published; and agreed to be accountable for all aspects of the work in ensuring that questions related to the accuracy or integrity are appropriately investigated and resolved.

## Conflict of Interest Statement

The author has nothing to disclose. The author declares that the research was conducted in the absence of any commercial or financial relationships that could be construed as a potential conflict of interest.
